# Epithelial Splicing Regulatory Protein (ESPR1) Expression in an Unfavorable Prognostic Factor in Prostate Cancer Patients

**DOI:** 10.3389/fonc.2020.556650

**Published:** 2020-10-26

**Authors:** Hyung Ho Lee, Andy Jinseok Lee, Weon Seo Park, Jongkeun Lee, Jongkeun Park, Boram Park, Jae Young Joung, Kang Hyun Lee, Dongwan Hong, Sung Han Kim

**Affiliations:** ^1^ Department of Urology, Center for Prostate Cancer, National Cancer Center, Goyangsi, South Korea; ^2^ Bioinformatics Analysis Branch, Research Institute, National Cancer Center, Goyangsi, South Korea; ^3^ Department of Pathology, National Cancer Center, Goyangsi, South Korea; ^4^ Department of Medical Informatics, College of Medicine, The Catholic University of Korea, Seoul, South Korea; ^5^ Biometrics Research Branch and Biostatics Collaboration Unit, Research Institute, National Cancer Center, Goyangsi, South Korea; ^6^ Department of Biomedicine & Health, Catholic University Graduate School, Seoul, South Korea

**Keywords:** biochemical recurrence (BCR), clinical validation, ESRP1 gene, prostate cancer, prostate cancer-specific mortality, survival analysis

## Abstract

**Background:**

To evaluate the role of epithelial splicing regulatory protein 1 (ESRP1) expression in survival prognoses and disease progression for prostate cancer (PC) using The Cancer Genome Atlas (TCGA) dataset and to validate it using patients’ prostatectomy specimens.

**Methods:**

A preliminary investigation into the clinical significance of ESRP1 in PC was conducted using TCGA PC PRAD dataset and then using immunohistochemistry in 514 PC patients’ tissue microarrays of radical prostatectomy specimens. The interpretation of immunohistochemistry was done using its intensity (high vs. low) or the semi-quantitative expression value (H-score, 0–300). The prognostic significance of ESRP1 expression was analyzed for biochemical recurrence (BCR), recurrence-free survival (RFS), overall survival (OS) and cancer-specific survival (CSS) using the Cox proportional-hazards model (p < 0.05).

**Results:**

In the publicly available prostate adenocarcinoma dataset, ESRP1 expression was significantly higher in the tumor samples compared to the normal samples (p < 0.001). Survival analysis showed that the tumor samples in the ESRP1-high group had significantly worse BCR-free survival and RFS compared to the ESRP1-low group (p < 0.05), whereas OS was not (p=0.08). These results were largely consistent with the 514 patients’ clinical data during a median 91.2 months of follow-up. After adjusting for significant prognostic clinicopathological factors, the multivariable models showed that the ESRP1 was a significantly risk factor for CSS (Hazard ratio 3.37, p = 0.034) and for BCR (HR 1.34, p=0.049) without any significance for OS (p=0.464).

**Conclusions:**

The higher ESRP1 expression appeared increased risk of disease progression and cancer-specific death in PC.

## Background 

Prostate cancer (PC) is the most common cancer among men aged 50 years and older. This genetic disease accounts for 15% of all cancers diagnosed in men worldwide, with over 1 million new cases diagnosed and approximately 307,000 deaths recorded in 2012 (1). The survival of patients with PC is reported to be over 90% when diagnosed in the early organ-confined stages but is 29% in metastasized cases in the United States (1). Therefore, there is an urgent need to identify biomarkers predictive of disease progression such as recurrence and metastasis for improving the survival of PC patients with metastatic disease.

PC progresses to a metastatic state by releasing PC cells into the systemic lymphatic and vascular tissues or by directly invading adjacent organs. Epithelial-mesenchymal transition (EMT) is a process by which cancer cells lose cell–cell adhesion and become motile, making it a necessary prelude to metastasis (2). During EMT, the RNA-binding protein epithelial splicing regulatory protein 1 (ESRP1) regulates the expression of epithelial cell-specific isoforms and causes a significant shift in expression from epithelial fibroblast growth factor receptor 2 (FGFR2)-IIIb to the mesenchymal FGFR2-IIIc splice variant (3). The association between ESRP1 expression and tumor progression has been demonstrated in many cancers including PC (4). Although the role of ESRP1 in metastasis has been reported in human prostatic tissue samples and in human PC cell lines (5). Though ESRP1 is known to be related to 17% of the early onset aggressive PC cases (6, 7). Accordingly, we sought to determine the clinical implications of ESRP1 mRNA expression using the publicly available prostate adenocarcinoma (PRAD) dataset from The Cancer Genome Atlas (TCGA). Based on the findings in the TCGA dataset, we validated the associations through survival analysis between two groups of patients with varying levels of ESRP1 expression based on immunohistochemistry (IHC) results in a radical prostatectomy (RP) tissue microarray from 514 PC patients at the National Cancer Center (NCC) of Korea.

## Methods

### Analysis of ESRP1 Gene Expression in the TCGA PRAD Dataset

The PRAD dataset from TCGA was used to conduct a preliminary investigation into the prognostic significance of *ESRP1* mRNA expression in PC (**Supplementary Figure 1**). Gene expression (2017-10-13 IlluminaHiSeq version) and clinical data (2016-04-27 version) of 550 PRAD samples were downloaded from Xena Browser (https://xenabrowser.net/). To determine whether *ESRP1* mRNA expression is correlated with survival outcomes, data on biochemical recurrence (BCR)-free survival, recurrence-free survival (RFS), and overall survival (OS) were compared between ESRP1-high (z-score ≥ 1.96) and ESRP1-low (z-score < 1.96) sample groups using log-rank tests. Further analysis on *ESRP1* using the TCGA PRAD dataset included the 2017-09-08 version of the copy number (called by GISTIC2 software), methylation, and protein expression data. Only samples with clinical, gene expression, copy number, and methylation data were used in this analysis (**Supplementary Figure 1A**).

The gene expression of TCGA PRAD were transformed by normalization method used in cbioportal (8). Z score of gene expressions was estimated by calculating the mean and variance of all samples with expression values. z-score = (raw expression value – mean(samples)) / standard deviation(samples). To classify ESRP1-high group and ESRP1-low group in Kaplan-Meier plot, gene expressions labeled with ESRP1-high were selected more than +1.96 * standard deviation from mean (0) and gene expressions labeled with ESRP1-low were selected less than -1.96 * standard deviation from mean (0), respectively

### Ethical Statement

All study protocols related to handling patient tissue samples and their clinicopathological information adhered to the ethical guidelines of the World Medical Association Declaration of Helsinki-Ethical Principles for Medical Research Involving Human Subjects. This study was approved by the Institutional Review Board (IRB) of the National Cancer Center Research Institute and Hospital (IRB No. NCCNCS05049). Given the retrospective nature of this study, written consent was waived by the approving IRB of the National Cancer Center Research Institute and Hospital.

### Patients and Tissue Samples

To validate the prognostic significance of ESRP1 expression, RP specimens from 514 PC patients at the NCC were used. These patients were diagnosed with PC between the years 2000 and 2015.

Of the 514 patients, 117 had received neoadjuvant androgen deprivation (NHT) prior to the RP. There were no missing clinicopathological data for any patient during the postoperative follow-up period of at least 6 months. All pathology results were reported according to the guidelines of the 2005 International Society of Urological Pathology (ISUP) consensus conference (9) and reviewed by a uropathologist with 30 years of experience (WSP) (**Supplementary Figure 1B**).

### Immunohistochemistry

**Figure 1:**
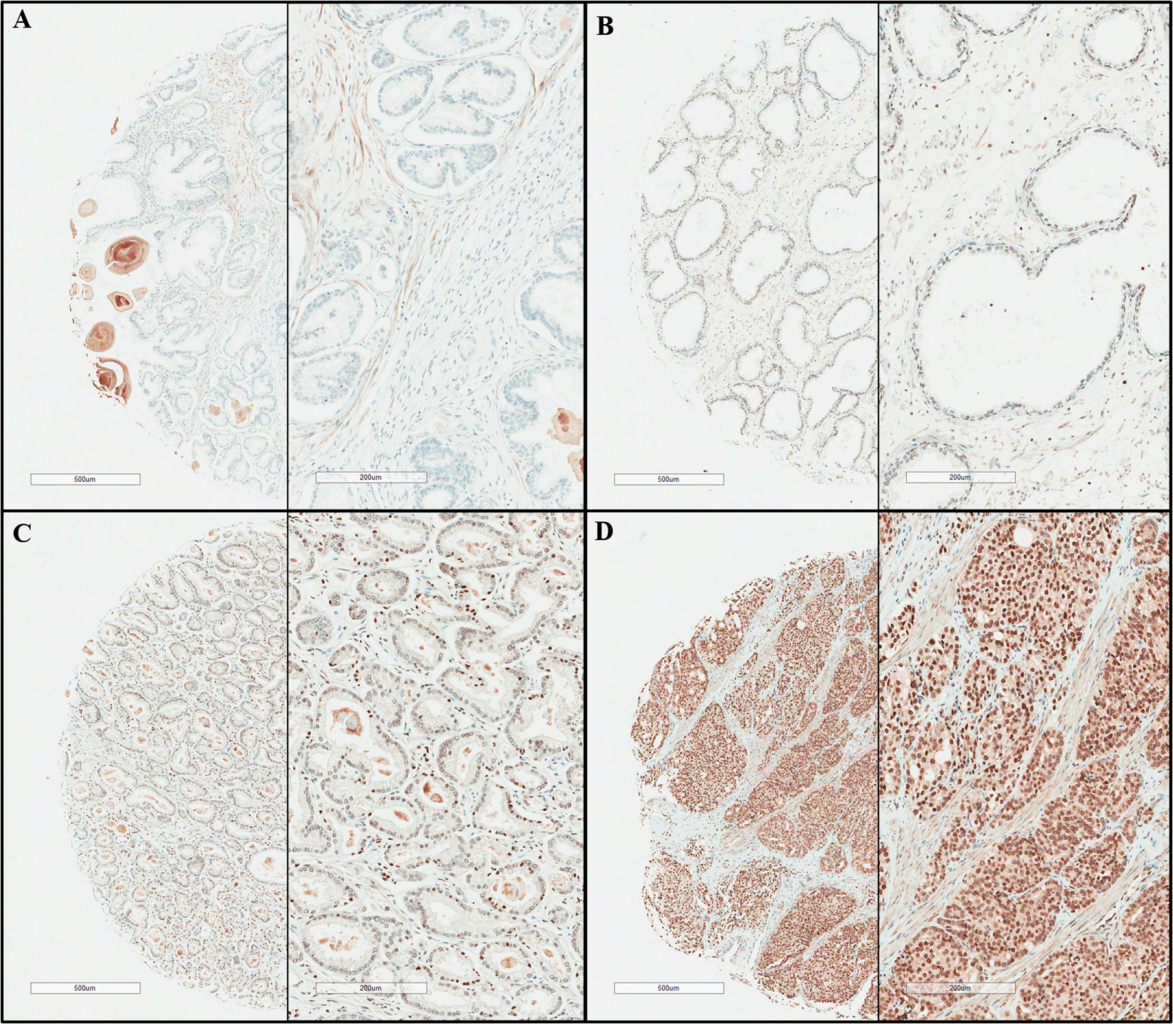
Presentative immunohistochemical staining results of epithelial splicing regulatory protein 1 (ESPR1). **(A)** negative, **(B)** weak positive, **(C)** moderate positive, **(D)** strong positive (original magnification x40, x100).

The tissue microarrays of the 514 prostatectomy specimens were prepared following the protocols described previously (10). TMA blocks were built using representative tumor areas and paired normal control tissue from formalin-fixed, paraffin- embedded tumor material and marked on standard hematoxylin/eosin (H&E)-stained sections for the expressions of tissue markers. The specimens were immunohistochemically stained for ESRP1 (Sigma-Aldrich), and the final score was determined from these two parameters as follows: negative (0), absence of ESRP1 staining in 100% of tumor cells; weak (1), intensity of 1+ in >70% of tumor cells or staining intensity of 2+ in 30% of tumor cells; moderate (2), intensity of 1+ in >70% of tumor cells, or staining intensity of 2+ in >30% but 70% of tumor cells, or staining intensity of 3+ in 30% of tumor cells; strong (3), intensity of 2+ in >70% of tumor cells, or staining intensity of 3+ in >30% of tumor cells. The negative (0) and weak (1) samples were considered as negative ESRP1 expression, whereas those with moderate (2) or strong (3) scores as positive ESFR1 expression, and the cases were identified pathologically by a senior uropathologist (WSP) blinded to the clinical outcome using the semi-quantitative H-score (0–300), including the intensity score (0 for negative, 1+ for weak, 2+ for moderate, and 3+ for strong) (**Figure 1**).

### Statistical Analysis

Association between each pathological feature and ESRP1 was investigated using Wilcoxon rank-sum test or Kruskal-Wallis test. Survival analysis was performed to examine the effects of these clinicopathological variables on BCR-free survival, OS, and cancer-specific survival (CSS). Patients with no event were censored at the last follow-up time. Survival curves were plotted using the Kaplan-Meier method and the log-rank test was used to compare survival between the high and low expression groups.

The continuous H-score was dichotomized based on a cut-off point acquired through the Contal and O’Quigley method (10). Both univariable and multivariable Cox proportional hazards models were performed to identify associations between ESRP1 expression and survival outcomes in BCR, OS, and CSS. Multivariable Cox proportional hazards models were constructed by adjusting for significant clinicopathological confounders. The Firth’s method was applied to handle sparse events as the hazard ratio from Cox proportional hazard model cannot be derived when there are no events. An overview of the analysis pipeline is provided in **Supplementary Figure 1**. Statistical analyses were performed with the SAS program (version 9.4; SAS Inc., Cary, NC, USA) and R package (version 3.3.3; http://www.R-project.org) with a statistically significance of two-tailed p-value < 0.05.

## Results

### Recurrence Analysis Based on the TCGA PRAD Data

Analysis of the tumor and normal tissues from the TCGA PRAD samples showed that *ESRP1* expression was significantly higher (p < 0.001) in the tumor samples than in the normal samples (**Supplementary Figure 2**). These results were largely consistent with the results obtained by the analysis of the TCGA-PRAD dataset (**Supplementary Figure 3** and **Supplementary Table 1**). The clinical dataset was collected from the prostate cancer cohort in TCGA. Clinical information including Age at initial pathologic diagnosis, OS, RFS, BCR, Gleason score, clinical stage(M), pathologic stage(N), pathologic stage(T), PSA value and history of neoadjuvant treatment were obtained from the clinical dataset. And our study excluded samples of no recodes (**Supplementary Table 1**). Survival analysis showed that the tumor samples in the *ESRP1*-high (z-score ≥ 1.96; n = 56) sample group had significantly worse BCR-free survival and RFS compared to those in the *ESRP1*-low (z-score < 1.96; n = 359) sample group (*p* < 0.05). OS, however, was not significantly different between the two groups (p=0.08) (**Supplementary Figure 3**).

### IHC Analysis of ESRP1 Expression in Human PC Tissues

Based on the preliminary findings from the analysis of the TCGA PRAD dataset, we hypothesized that *ESRP1* mRNA levels might be indicative of unfavorable prognosis in PC. To validate this clinical significance, the survival outcomes were compared between high and low levels of ESRP1 protein expression in the RP specimens of 514 PC patients obtained at the NCC of Korea. The baseline characteristics of these patients are summarized in **Table 1**. The mean H-score for ESRP1 expression from the 514 specimens was 247.5 ± 63.9, and 503 (97.9%) of the PC tissues stained positive for ESRP1. The Contal and O’Quigley method estimated a threshold of 270 to stratify the high and low ESRP1 expression groups. As a result, 245 (47.7%) and 269 (52.3%) samples were classified into the high and low ESRP1 expression groups, respectively. (**Figure 1**).

**Table 1:** Baseline characteristics (*n* = 514).

Characteristics	Number (%)
**Follow-up duration (months)**	
** Median (range)**	91.2 (1.4–179.7)
**Age (years)**	
** Mean ± sd**	65.4±7.1
**Prostate volume**	
** Median (range)**	31.0 (2.5–113.6)
**Tumor percentage**	
** Median (range)**	15.0 (0.0–95.0)
**PSA**	
** <3**	27 (5.3)
** 3–10**	251 (48.8)
** 10–20**	120 (23.4)
** ≥20**	116 (22.6)
**Neoadjuvant hormonal therapy**	117 (22.8)
**GS sum**	
** ≤6**	317 (61.7)
** 7**	141 (27.4)
** ≥8**	56 (10.9)
**T stage**	
** T2**	283 (55.3)
** T3**	112 (21.9)
** yT0-3**	117 (22.9)
**N stage**	
** N0**	384 (74.7)
** N1**	27 (5.3)
** Nx**	103 (20.0)
**Seminal vesicle invasion**	70 (13.6)
**Lymphatic infiltration**	52 (10.1)
**Perineural invasion**	277 (53.9)
**Margin positivity**	147(28.7)

PSA, prostate specific antigen; GS, Gleason score; std, standard deviation; range = min-max.

### High ESRP1 Expression Correlates With Unfavorable Prognosis

**Figure 2:**
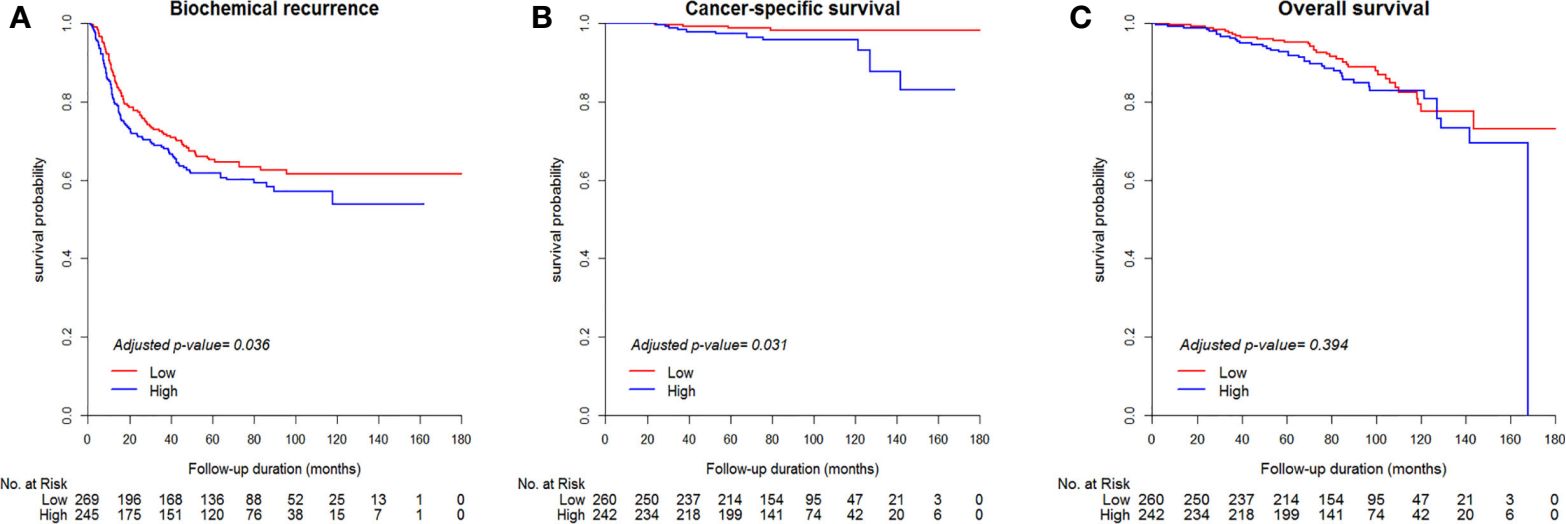
Kaplan-Meier survival curves of biochemical recurrence-free survival **(A)**, cancer-specific survival **(B)**, and overall survival **(C)** between the high (H-score > 270) and low (H-score ≤ 270) ESRP1 expression sample groups in the National Cancer Center cancer dataset. ESRP1, epithelial splicing regulatory protein 1.

The results of the univariable and multivariable Cox proportional hazards models are summarized in **Table 2**. In univariable model for baseline characteristics, BCR-free survival was significantly associated with age, prostate-specific antigen (PSA) level, Gleason score (GS), tumor percentage, NHT status, pathologic T (pT) stage, pathologic N (pN) stage, positive resection margin, perineural invasion status, seminal vesicle invasion status, and lymphovascular invasion status (p<0.05, **Supplementary Table 3**). Moreover, CSS was significantly associated with GS, tumor percentage, NHT status, pT stage, positive resection margin, lymphovascular invasion status, and seminal vesicle invasion status (p<0.05, **Supplementary Table 3**). Lastly, OS was significantly associated with age, tumor percentage, NHT status, pT stage, and seminal vesicle invasion status among other clinicopathological parameters (p<0.05, **Supplementary Table 3**). After adjusting for significant clinicopathological confounders, the multivariable Cox proportional hazards models showed that patients with high ESRP1 expression had a significantly higher risk of BCR (HR = 1.37, 95% CI = 1.02–1.83, p = 0.036) (**Figure 2A**) and worse CSS (HR = 3.43, 95% CI =1.12–10.54, p = 0.031) (**Figure 2B**); however, there was no association with OS (p>0.05)(**Table 2**) (**Figure 2C**). These results were largely consistent with the results obtained by the analysis of the TCGA-PRAD dataset (**Supplementary Figure 3** and **Supplementary Table 1**). The clinical dataset was collected from the prostate cancer cohort in TCGA. Clinical information including Age at initial pathologic diagnosis, OS, RFS, BCR, Gleason score, clinical M, pathologic N, pathologic T, PSA value and history of neoadjuvant treatment were obtained from the clinical dataset. And our study excluded samples of no recodes (**Supplementary Table 1**).

**Table 2:** Cox proportional hazard models for epithelial splicing regulatory protein 1 (ESRP1) protein expression (*n* = 514).

	ESRP1	Cut point	Number	Event (%)	Univariable		Multivariable	
					HR (95% CI)	p-value	HR (95% CI)	p-value
Biochemical recurrence (BCR)								
	continuous		514	191 (37.2)	1.002 (1.000–1.005)	0.0655	1.003 (1.000–1.006)	0.0271
	Low	≤270	269	93 (34.6)	1 (ref)		1 (ref)	
	High	>270	245	98 (40.0)	1.208 (0.910–1.605)	0.1915	1.367 (1.020–1.832)	0.0365
Overall survival (OS)								
	continuous		502	71 (14.1)	1.003 (0.999–1.008)	0.1392	1.002 (0.998–1.007)	0.3227
	Low	≤270	260	33 (12.7)	1 (ref)		1 (ref)	
	High	>270	242	38 (15.7)	1.288 (0.808–2.054)	0.2874	1.226 (0.768–1.956)	0.3935
Cancer specific survival (CSS)								
	continuous		502	17 (3.4)	1.047 (1.003–1.092)	0.0374	1.048 (1.005–1.092)	0.0281
	Low	≤270	260	4 (1.5)	1 (ref)		1 (ref)	
	High	>270	242	13 (5.4)	3.591 (1.171–11.018)	0.0254	3.432 (1.118–10.536)	0.0312

Adjusted for age, PSA, GS sum, tumor percent, NHT, T stage, N stage, lymphatic infiltration in the multivariate BCR model.

Adjusted for age, seminal vesicle in the multivariate OS model.

Adjusted for seminal vesicle in the multivariate CSS model.

### Genomic/Epigenomic Alterations and mRNA Expression of ESRP1 Gene Are Significantly Correlated

To study the underlying mechanism that correlates ectopic expression of ESRP1 and clinical significance, we investigated the copy number variation in *ESRP1* based on mRNA levels using the TCGA PRAD dataset. High *ESRP1* mRNA expression was significantly associated with higher copy number (p < 0.0001, **Supplementary Figure 4**). In particular, the median copy number of *ESRP1*-high samples was indicative of a gain, whereas *ESRP1* was diploid in most *ESRP1*-low samples.

Next, we sought to confirm whether the downregulation of the aforementioned EMT-related genes was related to methylation level. The *ESRP1*-high and *ESRP1*-low stratification further revealed that of the 30 CpG islands in ESRP1, 10 were significantly hypomethylated when *ESRP1* mRNA expression was high (p<0.05, **Supplementary Figure 5**).

### ESRP1 and EMT-Related Genes

The expression patterns of the EMT-related genes *SNAI1*, *SNAI2*, *CDH1*, *VIM*, *ZEB1*, *ZEB2* as well as those of *FGFR2* were analyzed using the TCGA PRAD dataset. The underlying mechanism among ESRP1 and EMT-related genes is shown in **Supplementary Figure 6**. The expression of five genes *SNAI2*, *CDH1*, *VIM*, *ZEB1*, and *ZEB2* was significantly lower in the *ESRP1*-high sample group than in the *ESRP1*-low sample group (p<0.05, **Supplementary Figure 6**). Furthermore, *ESRP1* expression showed a linearly increasing trend in concurrence with the 6 pathologic tumor stages (**Supplementary Figure 7**), suggesting that *ESRP1* mRNA expression is potentially correlated with tumorigenesis. N-cadherin and E-cadherin protein levels by *ESRP1* expression are shown in **Supplementary Figure 8**. The N-cadherin protein expression was significantly lower in the *ESRP1*-high group than in the *ESRP1*-low group, while E-cadherin protein expression was not significantly associated with *ESRP1* mRNA expression.

## Discussion

ESRP1 analysis in this study showed its potential as a prognostic biomarker for disease progression and cancer-specific death in PC, similar to the observation in the large-scale study by Gerhauser et al. (11). This study validated *ESRP1* expression using TCGA dataset and tissue specimens from 514 PC patients based on IHC and clinicopathological information. Our analytical methodology and identification of *ESRP1* as a potential biomarker for PC prognosis were similar to those of Gerhauser et al. who used tissues from 12000 patients and TCGA dataset (7). Our study and theirs suggested the ESRP1 gene as an independent risk factor of BCR and CSS, but not OS after validation with IHC of PC tissues and adjustment for several prognostic clinicopathological factors.

There were some differences between our study and that of Gerhauser et al regarding ESRP1. In the previous study, *ESRP1* was found to b ESRP1 gene as an independent risk factor of BCR and CSS, but not OS after validation with IHC of PC tissues and adjustment for several prognostic clinicopathological factors. ESRP1(H-score) related to a higher Gleason score and pT stage, whereas it only showed a significant correlation with pT stage and perineural invasion in this study (p=0.0114, **Supplementary Table 3**). Further, this study failed to show any significant relationship between ESRP1 and other known prognostic factors such as Gleason score, lymphovascular invasion, perineural invasion and pN stage (p>0.05). Our study and the previous study differed in terms of the baseline characteristics of the patient cohort and IHC interpretation. This study comprised 514 patients treated with radical prostatectomy including 117 patients treated with neoadjuvant hormone therapy and employed the semiquantitative H score (0–300) including the intensity (0, low, moderate, high) and expression percentage (0–100%). ESRP1 H-score showed low expression in neoadjuvant androgen deprivation therapy patient (yT0-3) compared to pT3 patients (p=0.0114, **supplementary Table 3**). In contrast, Gerhauser et al. evaluated the tumor profile of 12 000 patients based on genetic analysis and staining intensity in IHC and reported that *ESRP1* is a risk factor for BCR and CSS, which was similar to the findings of this study. Despite these differences, *ESRP1* was identified as a significant prognostic factor for PC in both studies. The interesting points about *ESRP1* are its progressive pathogenicity in disease progression and its potential role as a therapeutic target for FGFR inhibitors related to the FGFR signaling pathway (12, 13). Regarding the ability for pathogenetic progression, this study and the large-scale study by Gerhauser et al focused on EMT during cancer progression using TCGA dataset (7, 13). Genetic profiling analysis showed that the duplicated *ESRP1*—related to increased mRNA binding—was involved in EMT and that RNA splicing was found in 17% of the early onset PC cases and was found to be associated with aggressiveness and progression to metastasis (6). ESRP1 regulated alternative splicing in the epithelium and induced a change from an epithelial state promoting cell–cell adhesion to a mesenchymal state enabling invasive and migratory cell behavior (12–14).

As shown in **Supplementary Figure 6**, Epithelial splicing regulatory protein 1 (ESRP1) gene plays a role in regulating alternative splicing of FGFR2 gene. ESRP1 gene regulates the target genes such as SNAI1, SNAI2, ZEB1, ZEB2, CDH1, and VIM, by transferring signal to the EMT-related transcription factors depending on its isoform switching and is also known to be an important factor in deriving transitions to epithelial cells and mesenchymal cells (15). E-cadherin, and N-cadherin and vimentin are representative markers in epithelial cell and mesenchymal cell, respectively. They have positive correlation among expression of ESRP1, E-cadherin, and N-cadherin (**Supplementary Figure 8**) (16). In conclusion, ESRP1 is an important factor that directly affects genes that regulate the epithelial-mesenchymal transition (EMT) and promote prostate cancer invasion and metastasis (**Supplementary Figure 9**). Lu et al. also showed that in the transcriptome-wide remodeling of splicing regulation during metastasis, downregulation of the ESRP splicing network was a key feature of cancer cells with greater metastatic colonization potential (13). However, similar to other cancers, there are some controversies regarding the fact that ESRP expression in PC exhibits tumor-suppressing and tumor-enhancing capabilities in disease progression during EMT and functions as a predictor of both favorable and unfavorable survival outcomes (17–19). Jeong et al. pointed out that the discrepant findings of these previous studies may be suggestive of the plastic role of ESRP1 expression in tumor-specific tissues in view of the relation to FGF signaling pathways between *in vitro* and *in vivo* settings (12). However, this study and Gerhauser et al’s study showed that ESRP1 is an unfavorable factor in PC regardless of the PC type (early-onset, localized, or locally advanced) (7).

Another interesting point regarding *ESRP1* is its potential to function as a therapeutic target and monitoring candidate for FGFR inhibitors, chemotherapy, or radiation therapy. The FGF signaling pathway controls various processes in the normal cell cycle as well as the surrounding stroma, including the vasculature and is involved in oncogenesis and disease progression in many cancers, including PC (20). The net result of increased FGF signaling includes enhanced proliferation, resistance to cell death, increased motility and invasiveness, increased angiogenesis, enhanced metastasis, resistance to chemotherapy and radiation, and androgen independence, all of which could enhance tumor progression and clinical aggressiveness. Both *in vitro* and *in vivo* trials of FGF inhibitors in PC have demonstrated promising results (21–23), as FGF inhibitors targeting FGFRs or FGF signaling could directly affect both tumor cells and tumor angiogenesis. In particular, FGFRs could activate multiple signal transduction pathways that play a role in PC progression (18). Therefore, ESRP1 monitoring could help clinicians more accurately predict a patient’s prognostic outcome and develop the most effective personalized therapeutic strategy earlier.

**Figure 3:**
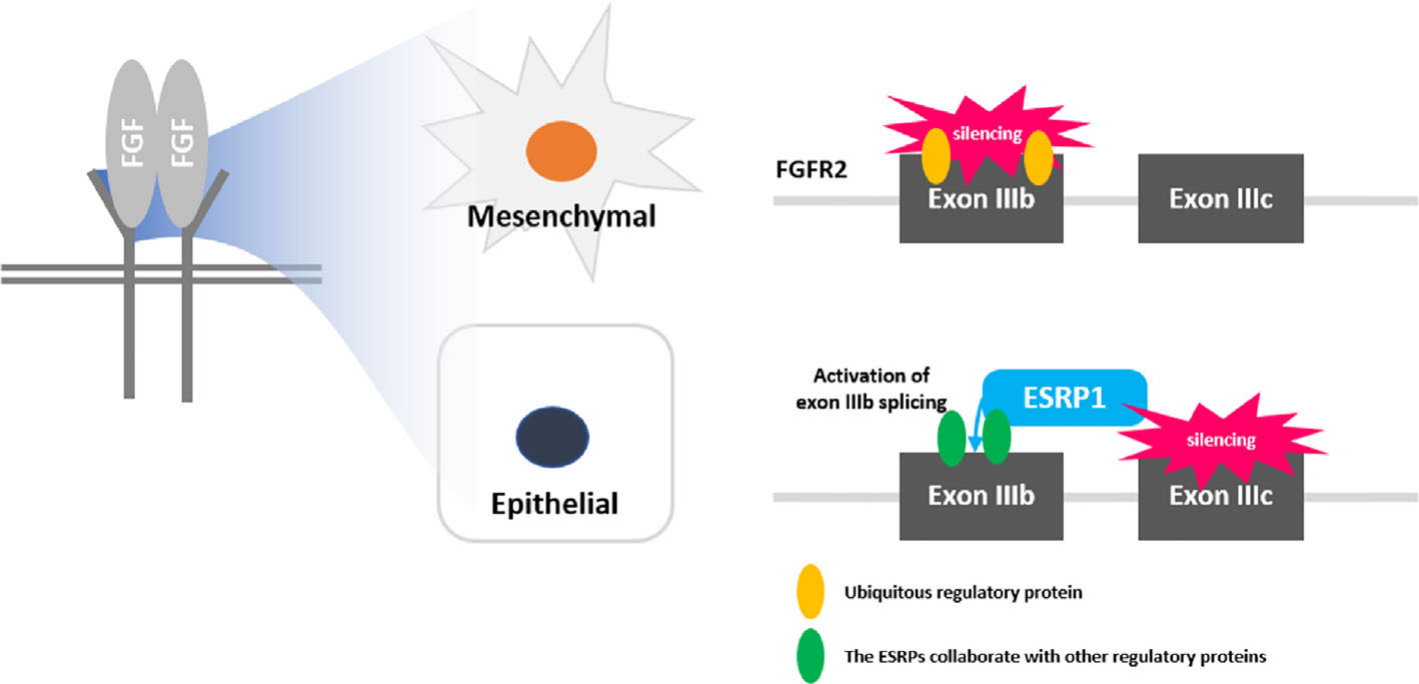
Schematic diagram of fibroblast growth factor receptor (FGFR) signaling and role of ESRP1 in epithelial-mesenchymal transition (EMT).

FGFR2, one of the four FGF receptors, regulates epithelial cell type-specific splicing program in conjunction with ESRP1 (12, 24). ESRP1-specific FGFR2 was directly associated with the differential processes of PI3K and MAPK pathways (25, 26). Specifically, through transcriptome profiling of PC cells and derivatives crossing the *in vitro* or *in vivo* barriers of metastasis, previous studies found several significant splicing factors that are differentially regulated during EMT, including ESRP1, ESRP2, and RBFOX2 (13, 27). Roca et al. also demonstrated that ESRP1 regulated mRNA splicing to induce metastasis using a PC mouse model (28). In another study using a mouse model induced with FGFR1 activation, EMT occurred in parallel with adenocarcinoma development (29). In addition, the ESRP1-specific FGFR2 was positively correlated with BCR and CSS, even after validation with PC specimens among the various FGFR types, similar to this study (13, 17). Therefore, it is now clear that maintaining the epithelium in the prostate is an important molecular mechanism for inhibiting tumorigenesis, and ESRP1 remodeling could be considered an integral regulatory process underlying metastasis, further suggesting ESRP1 as an important novel target gene for FGFR2 inhibitors to prolong survival and slow disease progression in PC (**Figure 3**).

This study had several limitations, including the potential for selection bias due to the retrospective design, postoperative short-term follow-up for PC-related death assessment, technical issues in the tissue microarray manufacturing and IHC procedures, and possible misinterpretation of the H-score of the IHC prostatectomy specimens. However, to our knowledge, there are only few clinical papers on the role of *ESRP1* in PC including NHT-PC. Overall, this study suggests that ESRP1 could serve as a therapeutic and prognostic target gene in PC and that gaining a better understanding of the role of ESRP1 in resistance against PC therapy and prognosis of PC could help to resolve this current clinical challenge.

## Data Availability Statement

The original contributions presented in the study are included in the article/**Supplementary Material**; further inquiries can be directed to the corresponding authors.

## Ethics Statement 

This study was approved by the Institutional Review Board (IRB) of the National Cancer Center Research Institute and Hospital (IRB No. NCCNCS05049). Given the retrospective nature of this study, written consent was waived by the approving IRB of the National Cancer Center Research Institute and Hospital.

## Author Contributions

(I) Conception and design: SHK and DWH. (II) Administrative support: HHL and SHK. (III) Provision of study materials or patients: JYJ, KHL, and SHK. (IV) Collection and assembly of data: JSL, JKL, JKP, WSP, and DWH. (V) Data analysis and interpretation: JSL, JKL, HHL, JKP, BRP, and SHK. All authors contributed to the article and approved the submitted version.

## Funding

This work was supported in part by grants from the National Cancer Center, Korea (NCC-18100211 and NCC-1810022 and NCC-1610450 and NCC-1711280), the National Research Foundation of Korea (NRF) grant funded by the Korea government (MSIT) (NRF-2019R1A2C1091023) and the Catholic Medical Center Research Foundation made in the program year of 2020 (for DWH).

## Conflict of Interest

The authors declare that the research was conducted in the absence of any commercial or financial relationships that could be construed as a potential conflict of interest.
