# Supplementary material for: Epithelial Splicing Regulatory Protein (ESPR1) Expression in an Unfavorable Prognostic Factor in Prostate Cancer Patients
Source: Front Oncol. 2020 Oct 26;10:556650. doi: 10.3389/fonc.2020.556650 (PMC7649424; doi:10.3389/fonc.2020.556650)
Supplement: Supplementary file 1 [file DataSheet_1.pdf]

SUPPLEMENTARY INFORMATION

Epithelial splicing regulatory protein (ESPR1) expression in an unfavorable prognostic factor in prostate cancer patients

Hyung Ho Lee[1], Andy Jinseok Lee[2], Weon Seo Park[3], Jongkeun Lee[2], Jongkeun Park[4], Boram Park[5], Jae Young Joung[1], Kang Hyun Lee[1], Dongwan Hong[2,6*+] and Sung Han Kim[1*+]

[1]Department of Urology, Center for Prostate Cancer, National Cancer Center, Goyangsi, Gyeonggi-do, 10408, Republic of Korea

[2]Bioinformatics Analysis Branch, Research Institute, National Cancer Center, Goyangsi, Gyeonggi-do, 10408, Republic of Korea

[3]Department of Pathology, National Cancer Center, Goyangsi, Gyeonggi-do, 10408, Republic of Korea

[4]Department of Medical Informatics, College of Medicine, The Catholic University of Korea, 222 Banpodae-ro, Seocho-gu, Seoul, 06591, Republic of Korea

[5]Biometrics Research Branch and Biostatics Collaboration Unit, Research Institute, National Cancer Center, Goyangsi, Gyeonggi-do, 10408, Republic of Korea

[6]Department of Biomedicine & Health, Catholic University Graduate School, 222 Banpodae-ro, Seocho-gu, Seoul, 06591, Republic of Korea

Table of contents

Supplementary Figures

Suppl. Figure 1 Schematic diagram of data analysis flow 3,4

Suppl. Figure 2 *ESRP1* gene expression between normal and tumor samples in the TCGA PRAD dataset 5

Suppl. Figure 3 Analysis of biochemical recurrence free survival, recurrence-free survival, and overall survival of TCGA PRAD dataset 6

Suppl. Figure 4 ESRP1 copy number variation in TCGA PRAD dataset 7

Suppl. Figure 5 Methylation in ESRP1 gene in TCGA PRAD dataset 8

Suppl. Figure 6 Correlations between expression of epithelial-mesenchymal transition related genes and ESRP1 gene expression in TCGA PRAD dataset 9

Suppl. Figure 7 *ESRP1* mRNA expression level by pathologic tumor stage. 10

Suppl. Figure 8 N-Cadherin and E-Cadherin protein levels by *ESRP1* expression level 11

Suppl. Figure 9 The regulation mechanism of ESRP1 during the epithelial to mesenchymal transition (EMT). 12

Supplementary Tables

Suppl. Table 1. Baseline characteristics from the prostate cancer cohort in TCGA PRAD dataset 13

Suppl. Table 2 Association between ESRP1 and clinicopathological parameters (NCC, n=514) 14

Suppl. Table 3 Univariable Cox proportional-hazards models for baseline characteristics (NCC, n=514) 15,16

Supplementary Figures

A

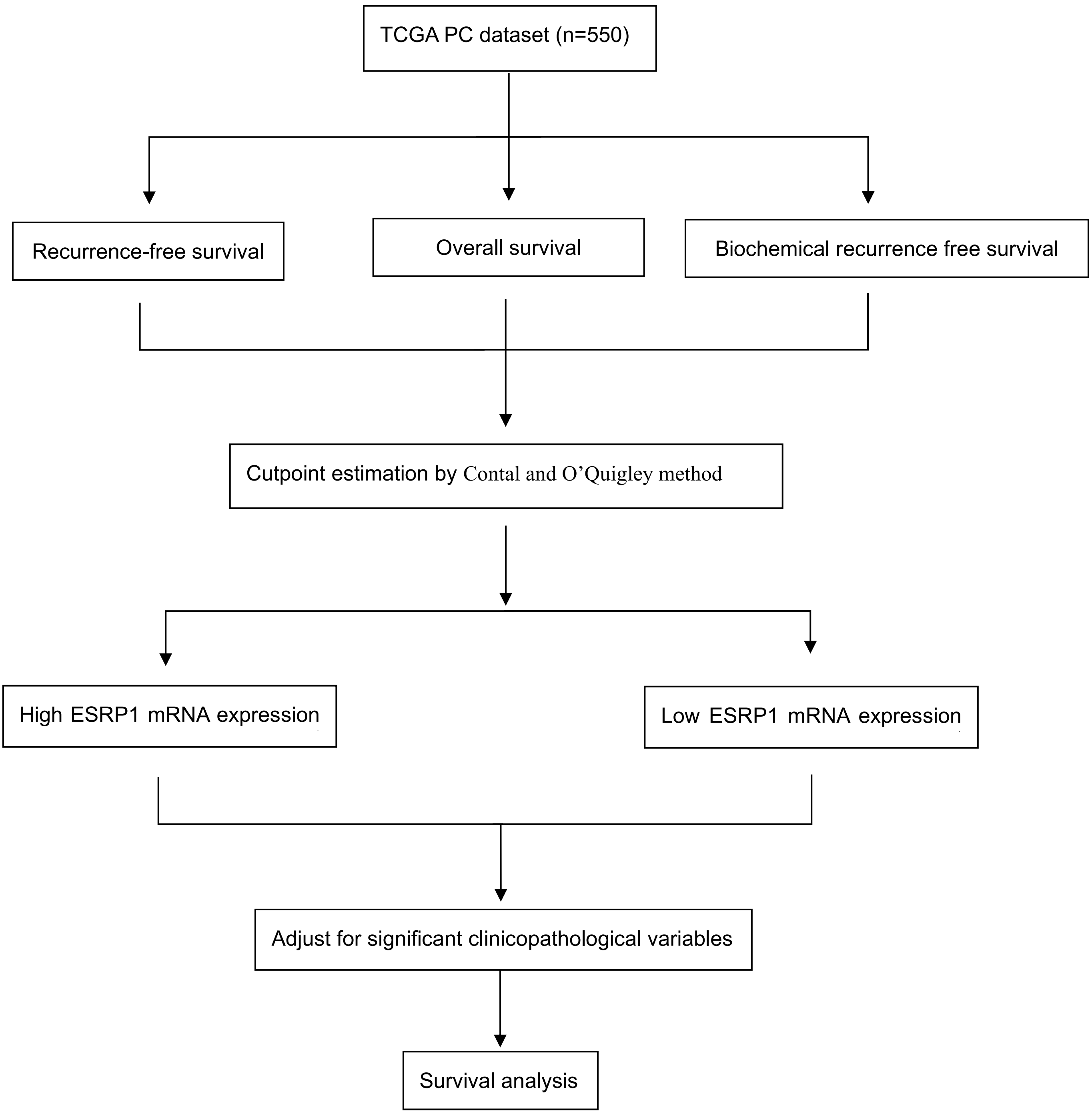

B

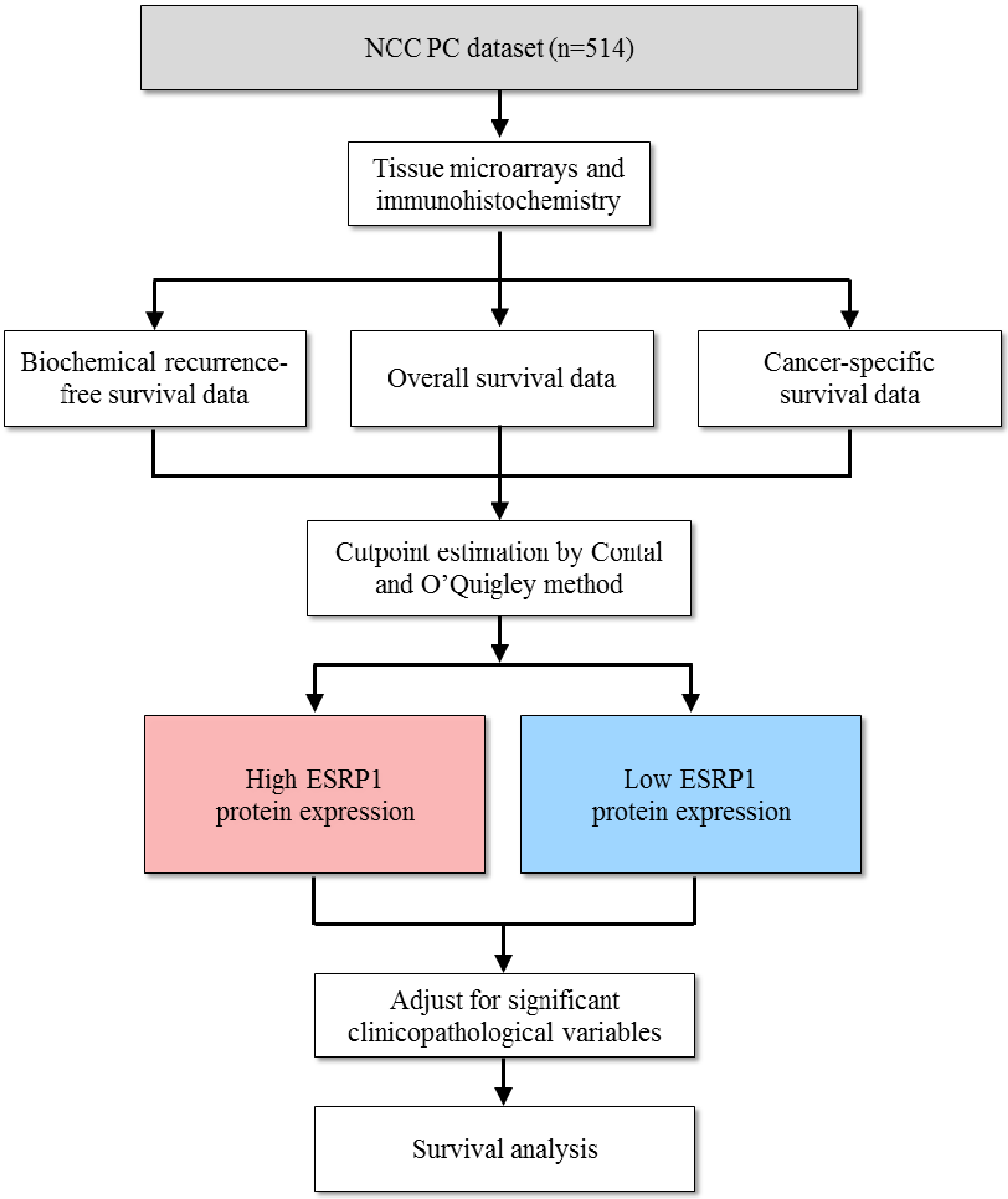

Supplementary Figure 1. A schematic diagram of the data analysis flow. To discover the candidate markers, mRNA expression, immunohistochemistry, clinical information and survival data of the TCGA (A) and NCC (B) PC datasets were analyzed, respectively.

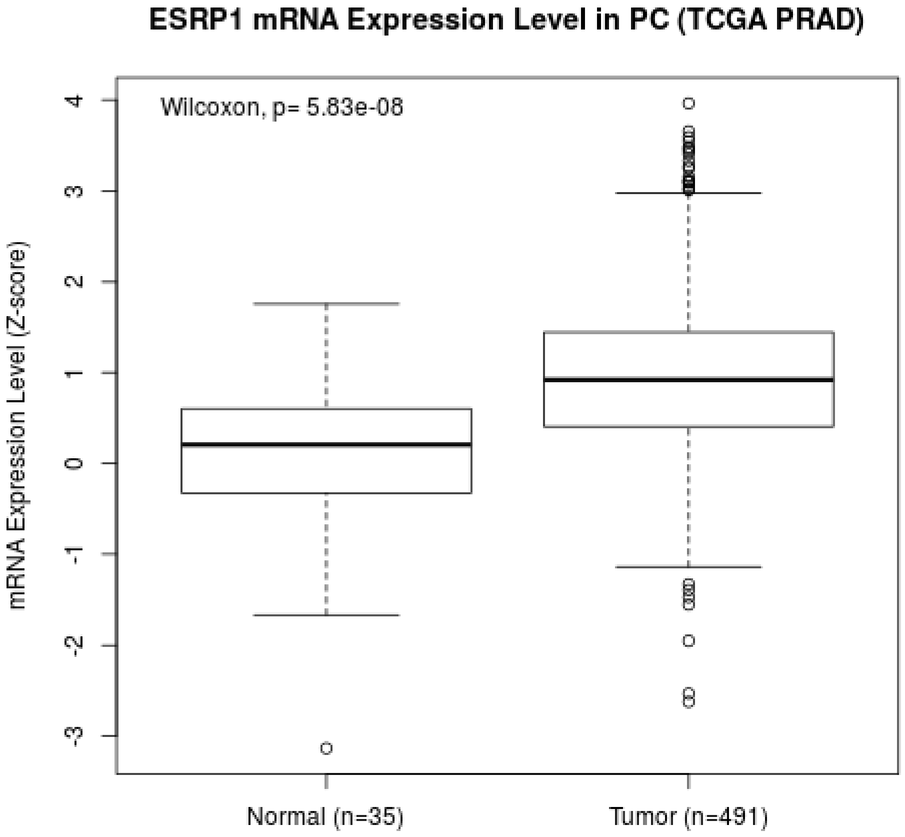

Supplementary Figure 2. *ESRP1* gene expression between normal and tumor samples in the TCGA PRAD dataset. The number of normal samples and tumor samples that had clinical, gene expression, copy number and methylation data were 35 and 491, respectively. The statistical test was calculated using two-sided Mann Whitney Wilcoxon test. The p-value was 5.83e-08.

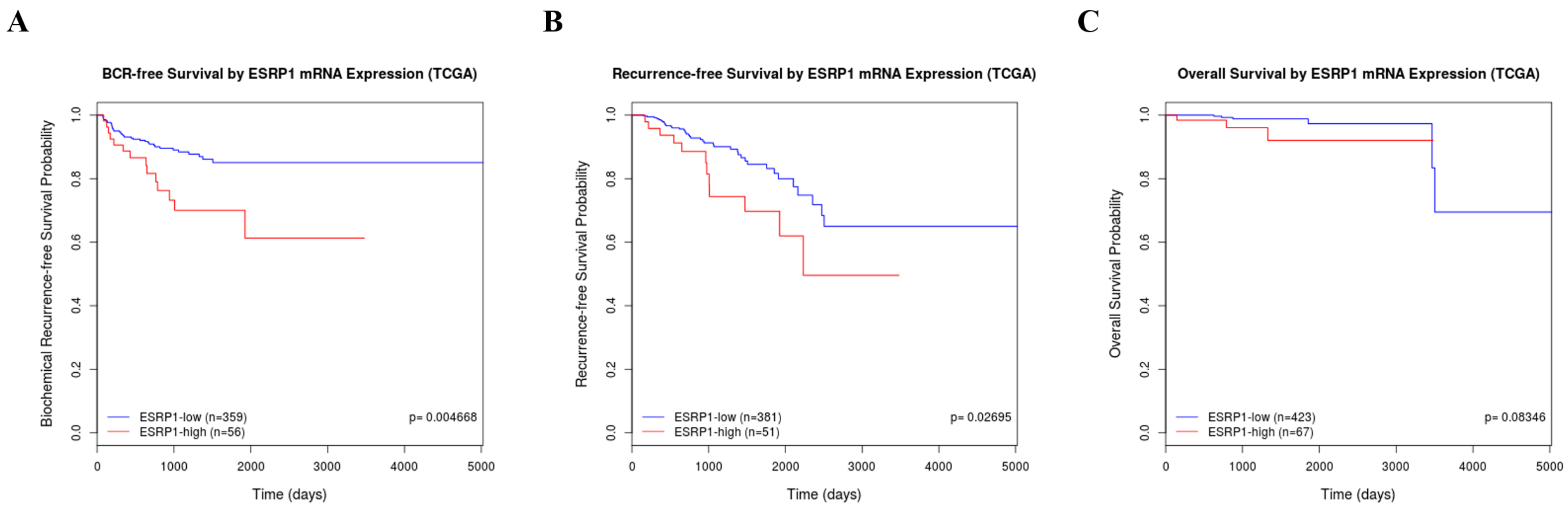

Supplementary Figure 3. Survival analysis of biochemical recurrence free survival, recurrence-free survival and overall survival of TCGA PRAD patients grouped by *ESRP1* mRNA expression level. Kaplan-Meier plot shows the biochemical recurrence free survival (A), recurrence-free survival (B) and overall survival (C) between the ESRP1-high (red) and the ESRP1-low (blue) sample groups. The log-rank p-values of the analyses A, B, and C were 0.004668, 0.02695, and 0.08346, respectively.

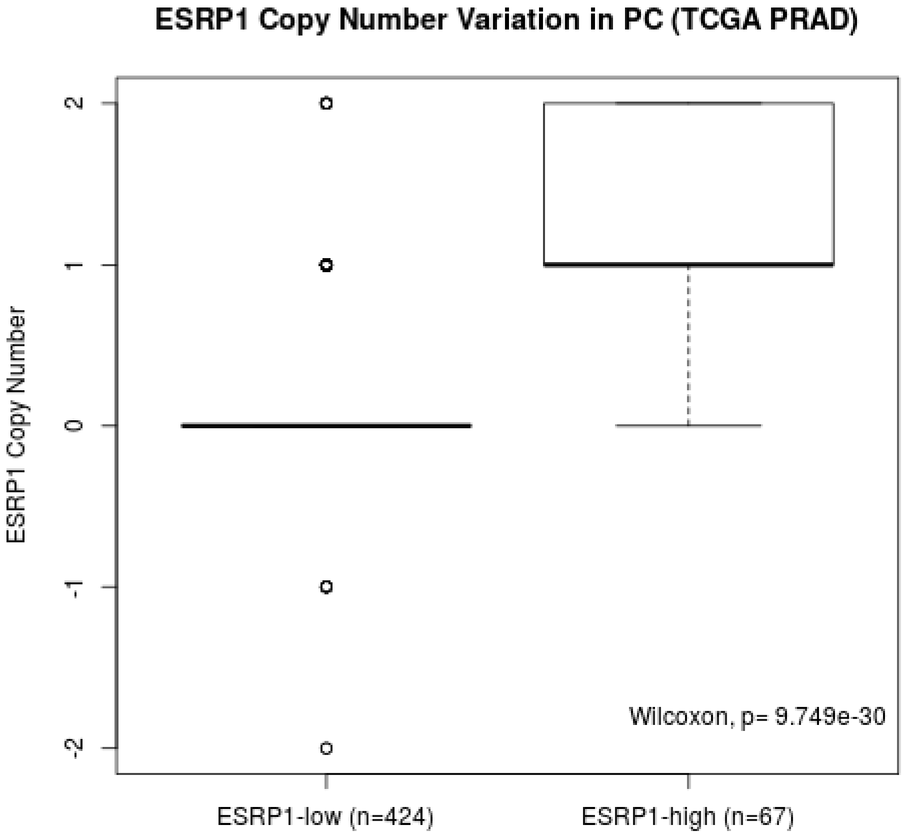

Supplementary Figure 4. *ESRP1* copy number variation in TCGA PRAD dataset. ESRP1-high samples had significantly higher ESRP1 copy numbers compared to the ESRP1-low samples (p-value = 9.749e-30; two-sided Mann Whitney Wilcoxon test).

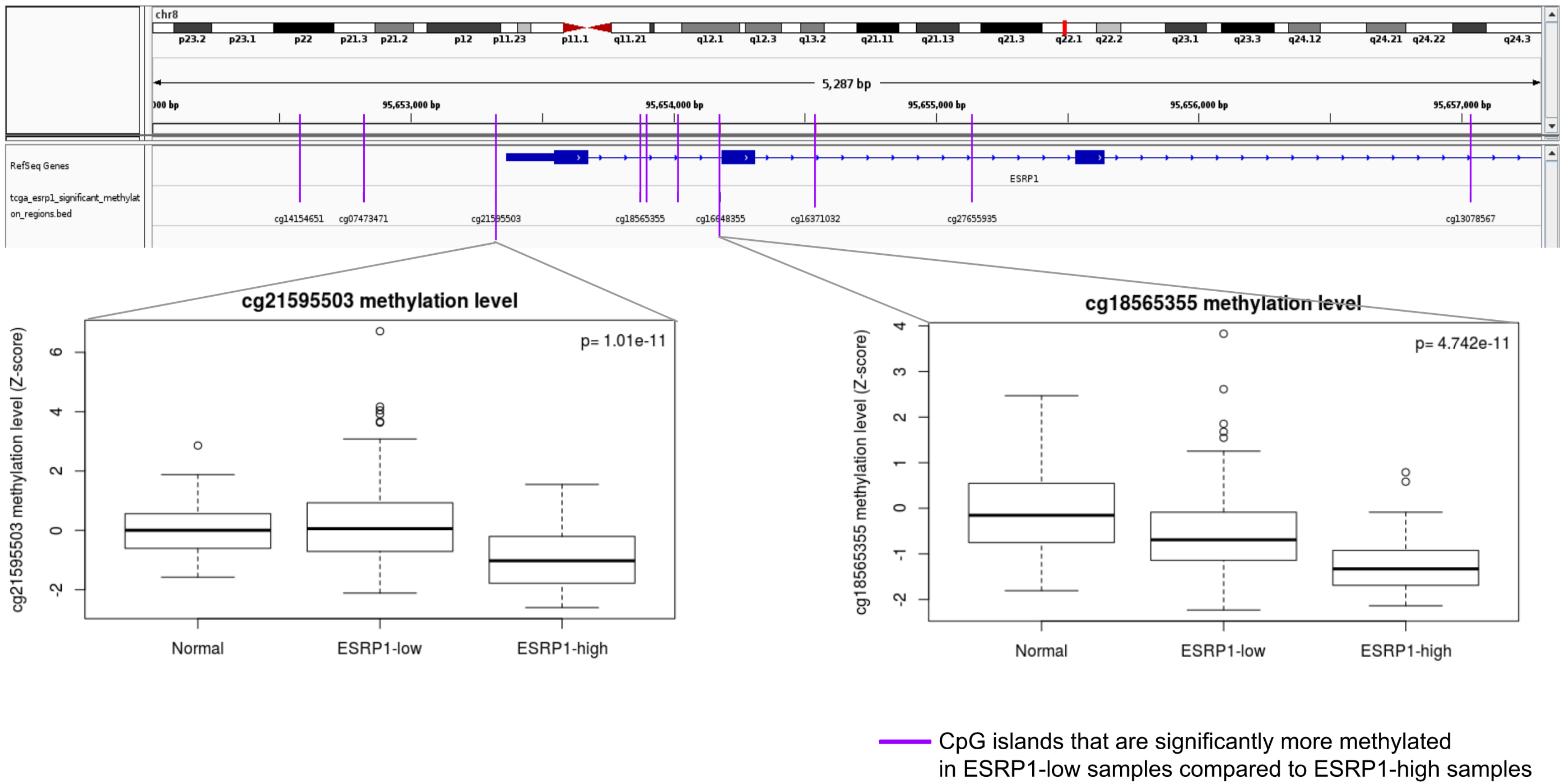

Supplementary Figure 5. Methylation level of CpG islands in ESRP1 genomic positions (chr8, 95,653,302 - 95,719,694; hg19) grouped by ESRP1-low and ESRP1-high sample groups (TCGA PRAD dataset). Of the 30 CpG islands in *ESRP1*, methylation level in 10 sites were significantly lower in the ESRP1-high sample group compared to the ESRP1-low sample group (p-value < 0.05; two-sided Mann Whitney Wilcoxon tests).

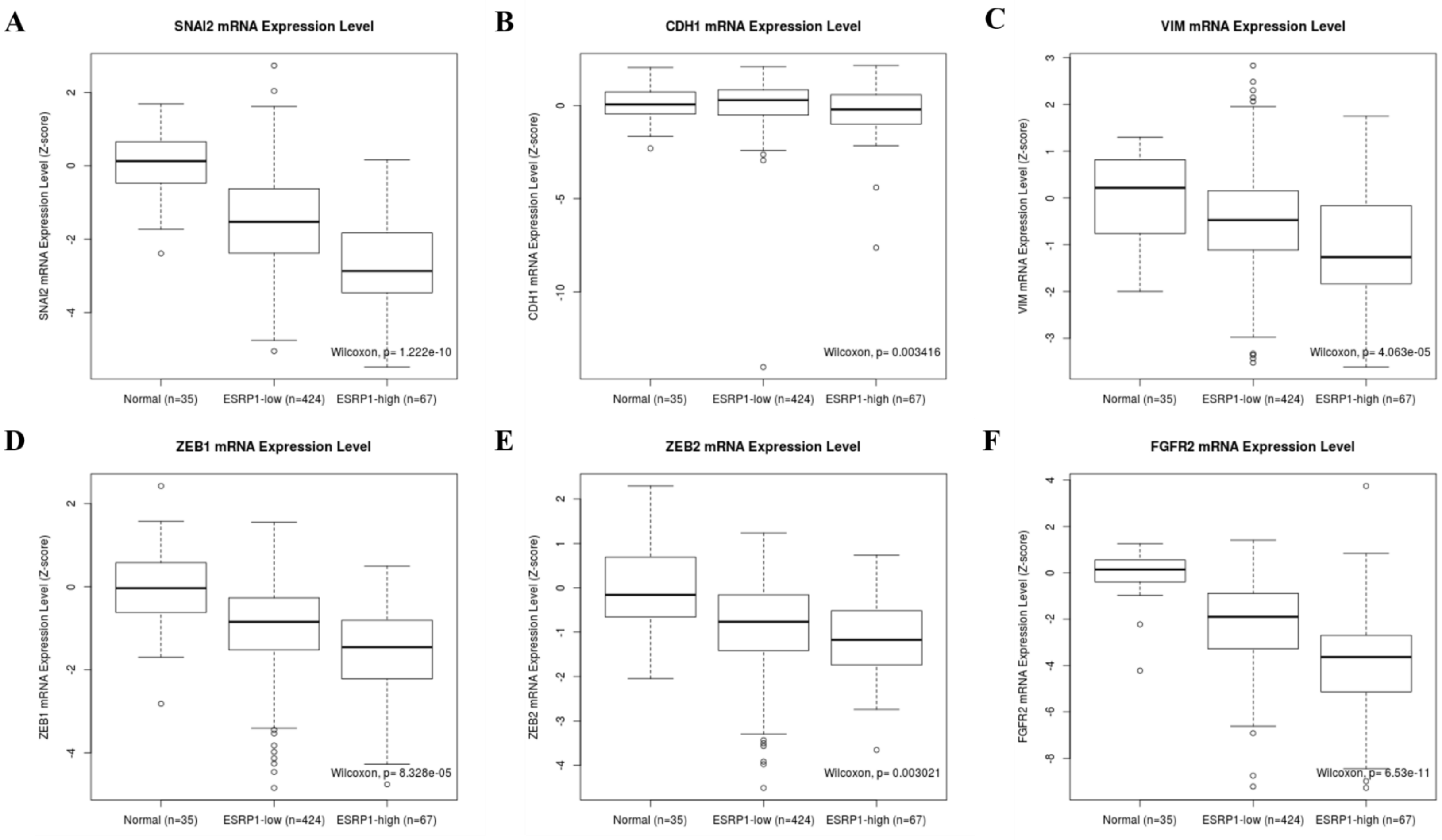

Supplementary Figure 6. Correlations between epithelial-mesenchymal transition (EMT) related genes and *ESRP1* expression in PC (TCGA PRAD). Consistent with previous findings in ovarian cancer, the 5 EMT-related genes (SNAI2, CDH1, VIM, ZEB1, ZEB2) are significantly downregulated in ESRP1-high tumor samples (two-sided Mann Whitney Wilcoxon tests). FGFR2, on the other hand, is generally downregulated in prostate cancer compared to the normal tissues and is significantly downregulated in the ESRP1-high sample group.

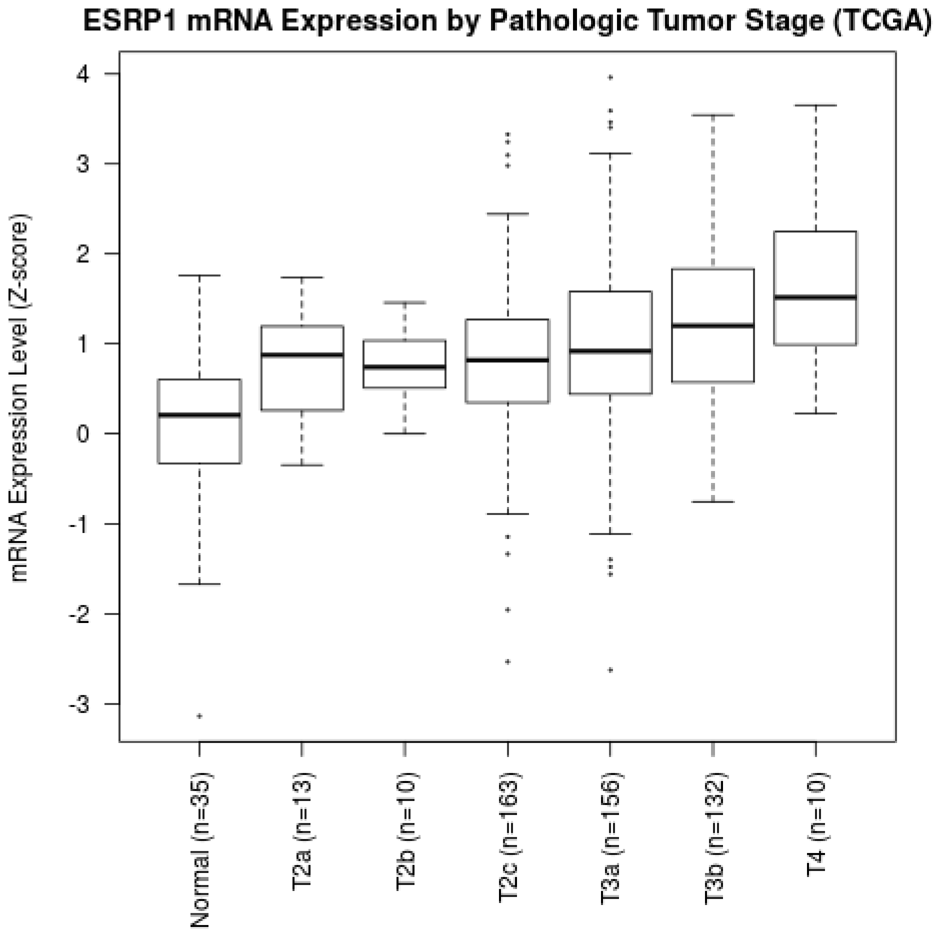

Supplementary Figure 7. *ESRP1* mRNA expression level by pathologic tumor stage.

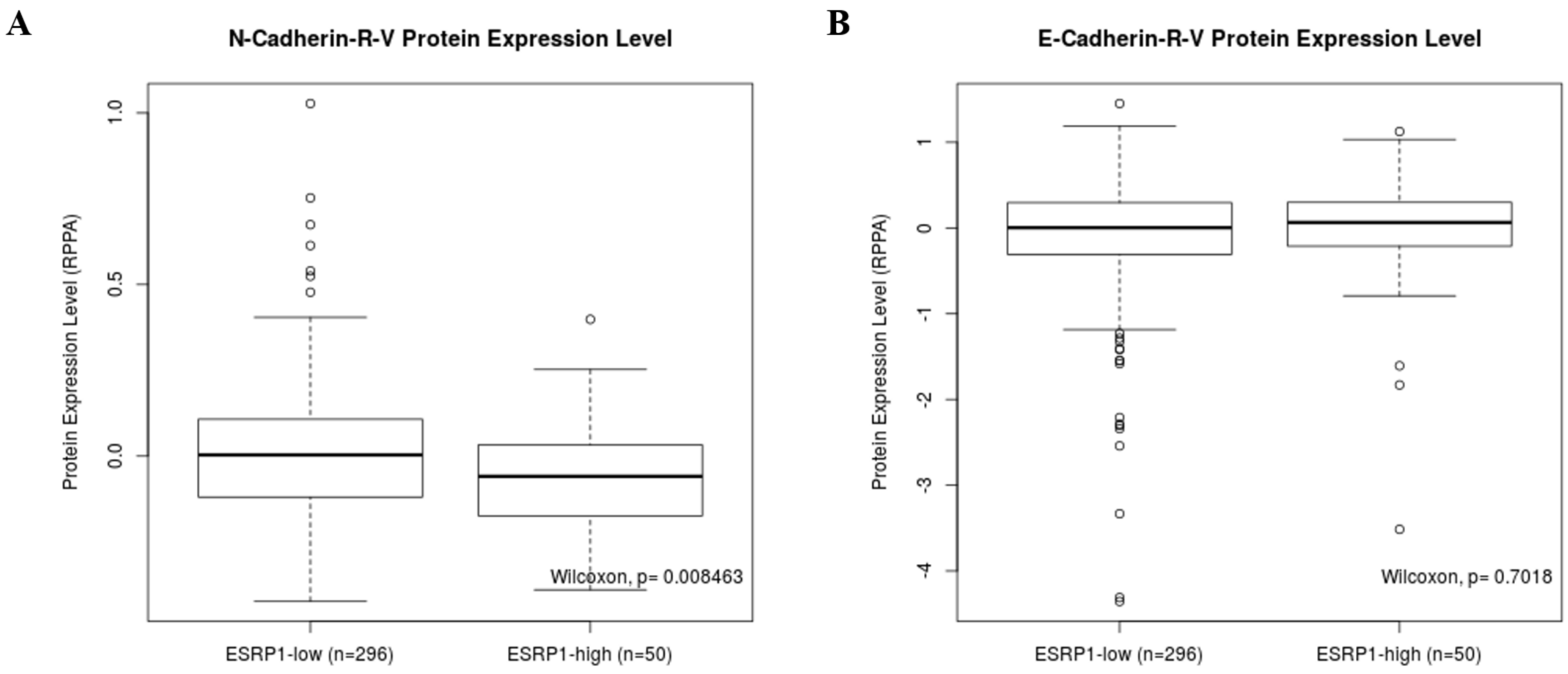

Supplementary Figure 8. N-Cadherin and E-Cadherin protein levels by *ESRP1* expression level. N-Cadherin expression was significantly lower in the ESRP1-high sample group compared to the ESRP1-low sample group (p-value = 0.008463; two-sided Mann Whitney Wilcoxon test), while E-Cadherin expression was not significantly different between the two groups.

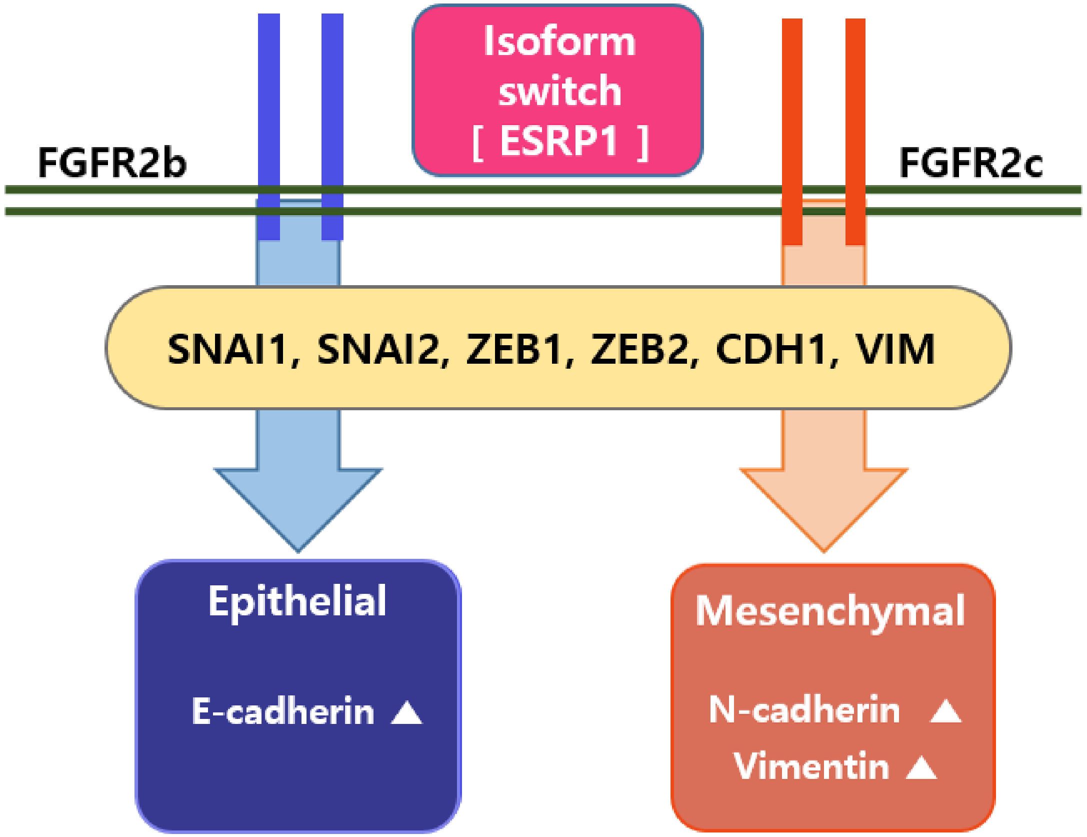

Supplementary Figure 9. The regulation mechanism of ESRP1 during the epithelial to mesenchymal transition (EMT). Switching of ESRP1 isoform regulates FGFR2b and FGFR2c type. FGFR2b (up-regulation of ESRP1) and FGFR2c (down-regulation of ESRP1) related with E-cadherin and N-cadherin, respectively.

Supplementary Tables

Supplementary Table 1. Baseline characteristics from the prostate cancer cohort in TCGA

		Patients [median (min~Max), %]
Age at initial pathologic diagnosis(years)		61 (41~78)
PSA (		0.1 (0~323)
Gleason score	≤6	9.05%
	7	49.70%
	≥8	41.25%
Clinica stage (M)	M0	99.34%
	M1	0.66%
Pathologic (N)	N0	81.37%
	N1	18.63%
Pathologic (T)	T2	38.16%
	T3	59.80%
	T4	2.04%

	status (%)		Duration [median (min~Max)] (days)
OS	Alive	97.98%	926 (23~5024)
	Dead	2.02%	
RFS	Alive	88.07%	866 (23~5024)
	Dead	11.93%	
BCR	Non-recurrence	87.41%	873 (0~5024)
	Recurrence	12.59%	

Supplementary Table 2. Associations between ESRP1 and clinicopathological parameters (NCC, *n* = 514)

		Number of samples (%)	ESRP1 H-score median (IQR)	p-value
GS sum	≤6	317 (61.7%)	270 (240-280)	0.1584
	7	141 (27.4%)	270 (250-280)	
	≥8	56 (10.9%)	280 (250-280)	
PSA	<10	278 (54.1%)	275 (250-280)	0.1314
	≥10	236 (45.9%)	270 (240-280)	
T stage	T2	283 (55.3%)	270 (250-280)	0.0114
	T3	112 (21.9%)	280 (250-280)	
	y0-3	117 (22.9%)	270 (230-280)	
N stage	N0-	384 (74.7%)	270 (250-280)	0.9996
	N1	27 (5.3%)	270 (250-280)	
	Nx	103 (20.0%)	270 (240-280)	
Lymphatic infiltration	No	462 (89.9%)	270 (240-280)	0.0742
	Yes	52 (10.1%)	280 (265-280)	
Perineural invasion	No	237 (46.1%)	270 (240-280)	0.1114
	Yes	277 (53.9%)	270 (250-280)	

P-value was calculated using Wilcoxon rank-sum test or Kruskal-Wallis test.

Supplementary Table 3. Univariable Cox proportional-hazards models for baseline characteristics (NCC, *n* = 514)

Characteristics		Number	Biochemical recurrence (BCR) (N=514, event=191)			Overall survival (OS) (N=502, event=71)			Cancer specific survival (CSS) (N=502, event=17)		
			event (%)	HR (95% CI)	p-value	event (%)	HR (95% CI)	p-value	event (%)	HR (95% CI)	p-value
Age		513	191 (37.2)	0.98 (0.96-1.00)	0.0131	71 (14.1)	1.10 (1.06-1.15)	<.0001	17 (3.4)	1.01 (0.94-1.08)	0.8457
PSA											
	<10	278	72 (25.9)	1 (ref)		34 (12.5)	1 (ref)		6 (2.2)	1 (ref)	
	≥10	236	119 (50.4)	2.42 (1.81-3.25)	<.0001	37 (16.2)	1.28 (0.80-2.05)	0.3069	11 (4.8)	2.03 (0.75-5.52)	0.1627
GS sum											
	≤6	317	82 (25.9)	1 (ref)		40 (12.9)	1 (ref)	(0.5259)	3 (1.0)	1 (ref)	
	7	141	71 (50.4)	2.48 (1.81-3.41)	<.0001	22 (16.2)	1.27 (0.75-2.14)	0.3765	8 (5.9)	5.93 (1.57-22.41)	0.0087
	≥8	56	38 (67.9)	3.86 (2.62-5.67)	<.0001	9 (16.1)	1.40 (0.68-2.90)	0.3601	6 (10.7)	11.07 (2.77-44.33)	0.0007
Tumor percent		511	191 (37.2)	1.02 (1.02-1.03)		71 (14.1)	1.02 (1.01-1.02)	0.0006	17 (3.4)	1.03 (1.02-1.05)	<.0001
NHT											
	No	397	128 (32.2)	1 (ref)		45 (11.6)	1 (ref)		9 (2.3)	1 (ref)	
	Yes	117	63 (53.9)	2.00 (1.48-2.70)	<.0001	26 (23.0)	2.06 (1.27-3.36)	0.0035	8 (7.1)	3.30 (1.26-8.61)	0.0149
T stage											
	T2	283	63 (22.3)	1 (ref)		27 (9.8)	1 (ref)	(0.0082)	2 (0.7)	1 (ref)	
	T3	112	65 (58.0)	3.62 (2.56-5.12)	<.0001	17 (15.3)	1.36 (0.74-2.51)	0.3207	7 (6.3)	7.41 (1.53-35.86)	0.0129
	yT0-3	117	63 (53.9)	3.15 (2.22-4.48)	<.0001	26 (23.0)	2.34 (1.36-4.01)	0.0021	8 (7.1)	9.91 (2.10-46.76)	0.0038
N stage											
	N0-	384	139 (36.2)	1 (ref)		55 (14.7)	1 (ref)	(0.2218)	13 (3.5)	1 (ref)	
	N1	27	16 (59.3)	1.77 (1.06-2.97)	0.0306	5 (19.2)	1.37 (0.55-3.44)	0.4971	2 (7.7)	2.06 (0.46-9.18)	0.3454

	Nx	103	36 (35.0)	0.95 (0.66-1.37)	0.7776	11 (10.9)	0.59 (0.30-1.17)	0.1331	2 (2.0)	0.43 (0.10-1.96)	0.2764
Seminal vesicle											
	No	444	142 (32.0)	1 (ref)		48 (11.1)	1 (ref)		7 (1.6)	1 (ref)	
	Yes	70	49 (70.0)	3.55 (2.56-4.93)	<.0001	23 (33.8)	3.45 (2.10-5.69)	<.0001	10 (14.7)	9.33 (3.55-24.55)	<.0001
Lymphatic infiltration											
	No	462	159 (34.4)	1 (ref)		60 (13.3)	1 (ref)		11 (2.4)	1 (ref)	
	Yes	52	32 (61.5)	2.49 (1.70-3.64)	<.0001	11 (22.0)	1.25 (0.65-2.41)	0.5051	6 (12.0)	3.54 (1.26-9.94)	0.0166
Perineural invasion											
	No	237	67 (28.3)	1 (ref)		25 (10.8)	1 (ref)		0 (0.0)	1 (ref)	
	Yes	277	124 (44.8)	1.84 (1.37-2.48)	<.0001	46 (17.0)	1.46 (0.90-2.38)	0.1299	17 (6.3)	27.56 (1.52-501.27)	0.0250
Margin											
	No	367	116 (31.6)	1 (ref)		46 (12.7)	1 (ref)		8 (2.2)	1 (ref)	
	Yes	147	75 (51.0)	1.90 (1.42-2.55)	<.0001	25 (17.7)	1.32 (0.81-2.15)	0.2690	9 (6.4)	2.73 (1.05-7.08)	0.0396

HR = hazard ratio; CI = confidence interval; NHT= neoadjuvant androgen deprivation; yT0-3=post neoadjuvant (radiation or systemic) therapy
